# Is Short-Course Antibiotic Therapy Suitable for *Pseudomonas aeruginosa* Bloodstream Infections in Onco-hematology Patients With Febrile Neutropenia? Results of a Multi-institutional Analysis

**DOI:** 10.1093/cid/ciad605

**Published:** 2023-10-05

**Authors:** Xiaomeng Feng, Chenjing Qian, Yuping Fan, Jia Li, Jieru Wang, Qingsong Lin, Erlie Jiang, Yingchang Mi, Lugui Qiu, Zhijian Xiao, Jianxiang Wang, Mei Hong, Sizhou Feng

**Affiliations:** State Key Laboratory of Experimental Hematology, National Clinical Research Center for Blood Diseases, Haihe Laboratory of Cell Ecosystem, Institute of Hematology and Blood Diseases Hospital, Chinese Academy of Medical Sciences and Peking Union Medical College, Tianjin, China; Tianjin Institutes of Health Science, Tianjin, China; Institute of Hematology, Union Hospital, Tongji Medical College, Huazhong University of Science and Technology, Wuhan, Hubei, China; State Key Laboratory of Experimental Hematology, National Clinical Research Center for Blood Diseases, Haihe Laboratory of Cell Ecosystem, Institute of Hematology and Blood Diseases Hospital, Chinese Academy of Medical Sciences and Peking Union Medical College, Tianjin, China; Tianjin Institutes of Health Science, Tianjin, China; State Key Laboratory of Experimental Hematology, National Clinical Research Center for Blood Diseases, Haihe Laboratory of Cell Ecosystem, Institute of Hematology and Blood Diseases Hospital, Chinese Academy of Medical Sciences and Peking Union Medical College, Tianjin, China; Tianjin Institutes of Health Science, Tianjin, China; State Key Laboratory of Experimental Hematology, National Clinical Research Center for Blood Diseases, Haihe Laboratory of Cell Ecosystem, Institute of Hematology and Blood Diseases Hospital, Chinese Academy of Medical Sciences and Peking Union Medical College, Tianjin, China; Tianjin Institutes of Health Science, Tianjin, China; State Key Laboratory of Experimental Hematology, National Clinical Research Center for Blood Diseases, Haihe Laboratory of Cell Ecosystem, Institute of Hematology and Blood Diseases Hospital, Chinese Academy of Medical Sciences and Peking Union Medical College, Tianjin, China; Tianjin Institutes of Health Science, Tianjin, China; State Key Laboratory of Experimental Hematology, National Clinical Research Center for Blood Diseases, Haihe Laboratory of Cell Ecosystem, Institute of Hematology and Blood Diseases Hospital, Chinese Academy of Medical Sciences and Peking Union Medical College, Tianjin, China; Tianjin Institutes of Health Science, Tianjin, China; State Key Laboratory of Experimental Hematology, National Clinical Research Center for Blood Diseases, Haihe Laboratory of Cell Ecosystem, Institute of Hematology and Blood Diseases Hospital, Chinese Academy of Medical Sciences and Peking Union Medical College, Tianjin, China; Tianjin Institutes of Health Science, Tianjin, China; State Key Laboratory of Experimental Hematology, National Clinical Research Center for Blood Diseases, Haihe Laboratory of Cell Ecosystem, Institute of Hematology and Blood Diseases Hospital, Chinese Academy of Medical Sciences and Peking Union Medical College, Tianjin, China; Tianjin Institutes of Health Science, Tianjin, China; State Key Laboratory of Experimental Hematology, National Clinical Research Center for Blood Diseases, Haihe Laboratory of Cell Ecosystem, Institute of Hematology and Blood Diseases Hospital, Chinese Academy of Medical Sciences and Peking Union Medical College, Tianjin, China; Tianjin Institutes of Health Science, Tianjin, China; State Key Laboratory of Experimental Hematology, National Clinical Research Center for Blood Diseases, Haihe Laboratory of Cell Ecosystem, Institute of Hematology and Blood Diseases Hospital, Chinese Academy of Medical Sciences and Peking Union Medical College, Tianjin, China; Tianjin Institutes of Health Science, Tianjin, China; Institute of Hematology, Union Hospital, Tongji Medical College, Huazhong University of Science and Technology, Wuhan, Hubei, China; State Key Laboratory of Experimental Hematology, National Clinical Research Center for Blood Diseases, Haihe Laboratory of Cell Ecosystem, Institute of Hematology and Blood Diseases Hospital, Chinese Academy of Medical Sciences and Peking Union Medical College, Tianjin, China; Tianjin Institutes of Health Science, Tianjin, China

**Keywords:** short course treatment, immunocompromised patients, *Pseudomonas aeruginosa*, bloodstream infection

## Abstract

**Background:**

Several studies have suggested that short-course antibiotic therapy was effective in *Pseudomonas aeruginosa* (PA) bloodstream infections (BSI) in immunocompetent patients. But similar studies in patients with hematological malignancies were rare.

**Methods:**

This cohort study included onco-hematology patients at 2 hematology centers in China. Inverse probability of treatment weighting was used to balance the confounding factors. Multivariate regression model was used to evaluate the effect of short-course antibiotic therapy on clinical outcomes.

**Results:**

In total, 434 patients met eligibility criteria (short-course, 7–11 days, n = 229; prolonged, 12–21 days, n = 205). In the weighted cohort, the univariate and multivariate analysis indicated that short course antibiotic therapy had similar outcomes to the prolonged course. The recurrent PA infection at any site or mortality within 30 days of completing therapy occurred in 8 (3.9%) patients in the short-course group and in 10 (4.9%) in the prolonged-course group (*P* = .979). The recurrent infection within 90 days occurred in 20 (9.8%) patients in the short-course group and in 13 (6.3%) patients in the prolonged-course group (*P* = .139), and the recurrent fever within 7 days occurred in 17 (8.3%) patients in the short-course group and in 15 (7.4%) in the prolonged-course group (*P* = .957). On average, patients who received short-course antibiotic therapy spent 3.3 fewer days in the hospital (*P* < .001).

**Conclusions:**

In the study, short-course therapy was non-inferior to prolonged-course therapy in terms of clinical outcomes. However, due to its biases and limitations, further prospective randomized controlled trials are needed to generalize our findings.

## Teaser

In this study, short-course antibiotic therapy was non-inferior to prolonged-course therapy in terms of clinical outcomes in part of onco-hematological patients with *Pseudomonas aeruginosa* (PA) bloodstream infections (BSI) and neutropenia febrile, with the potential added benefit of earlier hospital discharge.

## Graphical Abstract

**Graphical Abstract:**
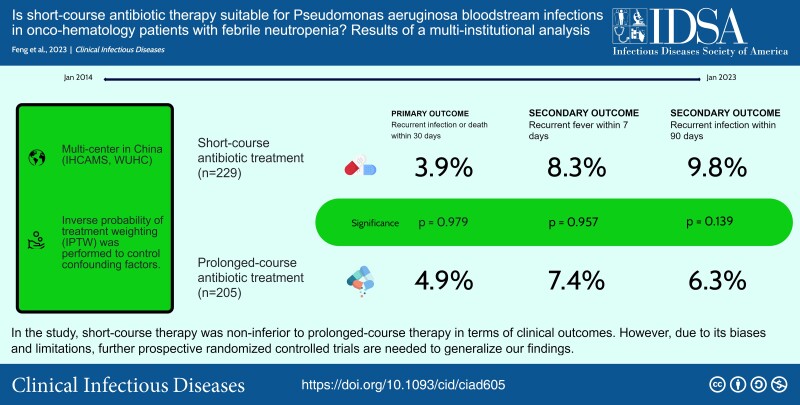
This graphical abstract is also available at Tidbit: https://tidbitapp.io/tidbits/is-short-course-antibiotic-therapy-suitable-for-pseudomonas-aeruginosa-bloodstream-infections-in-onco-hematology-patients-with-febrile-neutropenia-results-of-a-multi-institutional-analysis

Patients with hematological malignancies often experience neutropenia due to chemotherapy or allogenic hematologic stem cell transplantation (allo-HSCT) therapy, which increases their contact with the healthcare system and their use of antibiotics. This exposes them to a higher risk of antibiotic-related adverse events, such as resistance and toxicity. Therefore, reducing antibiotic exposure safely in this population is crucial. However, most randomized controlled trials (RCTs) investigating antibiotic durations either exclude or underrepresent immunocompromised patients. As a result, many clinicians are reluctant to prescribe short-course therapy for these patients. In the case of *Pseudomonas aeruginosa* (PA) bloodstream infections (BSI), it is widely accepted that more aggressive management is necessary compared to other gram-negative organisms, especially in the patients with hematological malignancies [1]. Although several studies have suggested that short-course antibiotic therapy does not increase the risk of infection recurrence or infection-related mortality [2–6], these findings lack reliability due to limitations such as a small proportion of immunocompromised patients, small sample sizes, and low utilization rates of short-course therapy [7].

In this study, we included a group of patients with hematological malignancies complicated with PA BSI after the inclusion and exclusion criteria. Using propensity score matching, we evaluated the safety and efficacy of short-course treatment in this populations, with infection-related mortality and recurrence as the primary outcomes.

## METHODS

### Study Population

All patients with hematological malignancies with a positive blood culture for PA admitted to 2 hospitals (Chinese Academy of Medical Sciences [IHCAMS], a 767-bed blood diseases hospital in Tianjin, Wuhan Union Hospital of China affiliated to Tongji Medical College of Huazhong University of Science and Technology [WUHC], a 420-bed blood diseases ward in Hubei) in China between January 2014 and January 2023 were evaluated. After visual inspection of the durations of targeted antibiotic therapy prescribed, short course was defined as 7–11 days of antibiotics, whereas prolonged course was defined as 12–21 days. STROBE guidelines for reporting in epidemiological studies were followed for the reporting of the current study. This study was approved by the ethical committee of the IHCAMS, the WUHC.

**Figure 1.**
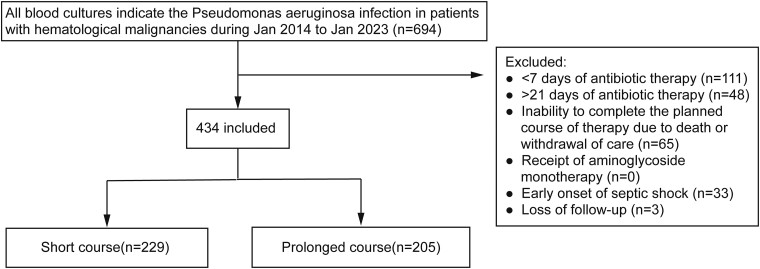
Study population.

Patients who met any of the following conditions were excluded (Figure 1): (1) receipt of less than 7 days of antibiotic therapy; (2) receipt of more than 21 days of antibiotic therapy; (3) inability to complete the planned course of therapy due to death or withdrawal of care; (4) receipt of aminoglycoside monotherapy during any portion of the treatment course; (5) had early onset of septic shock; (6) lost to follow-up within 90 days after stopping antibiotic therapy.

Of note, all patients with acute leukemia, lymphoma, multiple myeloma, and myelodysplastic syndrome (MDS) undergo guideline-directed chemotherapy and necessary HSCT. All patients received peripherally inserted central catheter (PICC) for the administration of chemotherapy drugs. In our center, routine antibiotic prophylaxis is not included in the protocol, even for neutropenic patients upon discharge. Definite treatment for PA infections is formulated based on the susceptibility testing of the initial blood culture. The indications for discontinuation of antibiotic therapy in our center: clinical improvement with resolution of infection-related signs and symptoms; negative blood cultures; successful control or removal of the identified source of infection (eg, central venous catheterization); resolution of infection-related manifestations, normalization of inflammatory markers, and neutropenia recovery [8] (absolute neutrophil count (ANC) > 0.5 × 10^9^ cells/L). Rare patients with persistent neutropenia but improving infection signs and controlled infection sources may stop antibiotics if their granulocytes are rising, even if not above 0.5 × 10^9^ cells/L, as per the clinicians’ decision.

### Outcomes

The primary outcome was a composite outcome that included recurrent PA infection or death, both within 30 days of discontinuing antibiotic therapy (patients unable to complete the whole course of antibiotic treatment due to death or withdrawal of care were excluded from the study, because they did not have the chance to be evaluated for the outcomes, that is, to limit the impact of immortal time bias [9]). The second outcome included recurrent PA infection within 90 days and recurrent fever within 7 days of discontinuing antibiotic therapy. Other endpoints observed included length of hospital stay. The recurrence of PA-BSI was defined as the subsequent isolation of PA from blood culture and presentation of infection related symptoms and signs within 90 days after stopping antibiotic therapy [10]. And recurrent fever was defined as recrudescent fever after previously meeting the criteria for defervescence, except for fever caused by other pathogens.

### Data Collection

Information regarding primary disease, management (chemotherapy, immunosuppression therapy, and allo-HSCT), tumor stage (induction, consolidation, and relapse/refractory disease), comorbidities (chronic liver disease, chronic renal disease, and diabetes mellitus), ANC at the onset of BSI, ANC at the day of the end of antibiotics (if not available, the ANC from the previous or subsequent day can be used as a substitute), duration of neutropenia before BSI, sources of infection and control measures, clinical severity (shock), clinical complications (pneumonia, oral mucositis, perianal mucositis, observed until the day 7 after the onset of BSI), microbiology data (MDR and CRPA), and antibiotic treatment regimen, including clinical outcomes, were collected by chart review for all patients. The criteria for BSI sources used in our study were based on the Centers for Disease Control and Prevention definitions for specific types of infections (https://www.cdc.gov/nhsn/pdfs/pscmanual/4psc_clabscurrent.pdf accessed January 2023) (Supplementary Table 1). Source control required device withdrawal or replacement in cases of urinary catheter, vascular catheter and biliary prosthesis infections, or adequate surgical or image-guided drainage in cases of surgical site infection, skin and soft tissue infection.

Tumor remission is judged according to the guidelines for the diagnosis and treatment of acute leukemia [11] and lymphoma [12, 13]. Immunosuppressive therapy is defined as receiving glucocorticoids and immunosuppressant drugs at the same hospitalization. Neutropenia was defined as an ANC < 0.5× 10^9^/L, and severe neutropenia was defined as an ANC < 0.1× 10^9^/L. Septic shock is characterized by systemic inflammatory response syndrome (SIRS) in the presence of a documented infection, leading to circulatory dysfunction, cellular and metabolic abnormalities, and organ dysfunction. Shock was defined as having a systolic pressure <90 mmHg, unresponsive to fluid treatment or requiring vasoactive drug therapy.

Adequately empirical therapy was defined as receiving 1 or more antimicrobial agents with in vitro activity within 48 hours of the onset of PA BSI, whereas the opposite is an inadequate empirical therapy (IET48h). Fever is defined as a core temperature (rectal) of 37.5°C–38.3°C (99.5°F–100.9°F), a skin temperature (axillary) >37.2°C. Defervescence was defined as a core temperature less than 37.5°C or a skin temperature (axillary) <37.2°C or for more than 24 hours.

### Microbiological Studies

Clinical samples were processed at the microbiology laboratory of each participating center in accordance with standard operating procedures. PA was identified using standard microbiological techniques at each center. In vitro susceptibility was determined according to the CLSI recommendations. We determined an isolate to be an MDR PA isolate when it was not susceptible to at least one agent in 3 or more of the following antimicrobial categories: aminoglycosides, antipseudomonal carbapenems, antipseudomonal fluoroquinolones, antipseudomonal cephalosporins, antipseudomonal penicillins plus-lactamase inhibitors.

### Statistical Analyses

Baseline categorical data were compared using the χ^2^ test, and continuous data were compared using the Wilcoxon rank sum test. To balance differences with respect to baseline characteristics between the 2 groups, inverse probability of treatment weighting (IPTW) was performed [14]. Covariates used for generating propensity scores included age, sex, primary hematological disease, chemotherapy in this hospitalization, allo-HSCT, immunosuppressive therapy, tumor stage, comorbidities (chronic liver disease, chronic renal disease, and diabetes mellitus), sources of infection, infection source control, shock, oral mucositis, perianal mucositis, ANC < 0.1× 10^9^/L on day 1 of BSI, duration of neutropenia before BSI, MDR-PA, CRPA, IET48h, combination regimen. Baseline characteristics were considered balanced if standardized mean difference (SMD) values were <10%. In the final analysis, odds ratios (ORs) and 95% confidence intervals (CIs) for the composite outcome were estimated using weighted regression, adjusting for variables with SMD greater than 10%. *P* value < .05 was considered statistically significant for all tests. Statistical analysis was completed using R version 4.2.

## RESULTS

The flowchart illustrating the study inclusion process is depicted in Figure 1. Among all patients with hematological malignancies, 59% (406 cases) had acute myeloid leukemia (AML), 24% (167 cases) had acute lymphoblastic leukemia (ALL), 86% of the patients underwent high-intensity chemotherapy, and 11.8% underwent allo-HSCT before developing BSI. Severe neutropenia affected over 50% of them (Supplementary Table 2). And the median neutropenia days in our cohort were 10 days (interquartile range [IQR], 4–16 days). Finally, of the 434 patients who met eligibility criteria, 229 (52.7%) received short-course therapy (median, 8 days; IQR, 8–10), and 205 (47.3%) received prolonged therapy (median, 15 days; IQR, 13–18). Clinical characteristics of the unweighted and weighted cohorts are shown in Table 1. Baseline clinical characteristics of the weighted cohort were well balanced. More than 85% of patients in the 2 groups received high-intensity chemotherapy during the same hospitalization:51.7% of patients were at the tumor remission state in the short-course group and 47.8% in the prolonged-course group. A small portion of patients who discontinued antibiotics before ANC reached 0.5 × 10^9^/L, but with an upward trend, were evenly distributed in the short-course group and the prolonged-course group. Comparing the clinical outcomes of the 2 groups, no difference was shown (Supplementary Table 3).

**Table 1.** Characteristics of Patients With Bloodstream Infections Due to PA

	Full Cohort				Weighted Cohort			
Characteristic	Short Course (n = 229, 52.7%)	Prolonged Course (n = 205, 47.3%)	*P* Value	SMD	Short Course (n = 205, 50.0%)	Prolonged Course (n = 205, 50.0%)	*P* Value	SMD
Age, median (IQR), y	39.0 (23.5–51.0)	42.0 (27.0–52.0)	.145	0.113	40.0 (25.5–52.0)	42.0 (27.0–52.0)	.498	0.077
Female (%)	101 (44.1)	96 (46.8)	.637	0.055	91 (44.4)	96 (46.8)	.692	0.049
Disease (%)								
ALL	48 (21.0)	61 (29.8)	.046	0.203	48 (23.4)	61 (29.8)	.302	0.111
AML	148 (64.6)	103 (50.2)	.003	0.294	125(61.0)	103 (50.2)	.037	0.217
Others	33 (14.4)	41 (20.0)	.156	0.149	32 (15.6)	41 (20.0)	.302	0.115
Tumor stage (%)								
Induction	64 (27.9)	56 (27.3)	.969	0.014	59 (28.8)	56 (27.3)	.826	0.033
Consolidation	125 (54.6)	98 (47.8)	.189	0.136	106 (51.7)	98 (47.8)	.489	0.078
Relapse/refractory disease	40 (17.5)	51 (24.9)	.076	0.182	40 (19.5)	51 (24.9)	.235	0.129
Chemotherapy (%)	202 (88.2)	171 (83.4)	.195	0.138	179 (87.3)	171 (83.4)	.328	0.111
Allo-HSCT, past 100 d (%)	26 (11.4)	33 (16.1)	.194	0.138	26 (12.7)	33 (16.1)	.399	0.097
Immunosuppressive therapy (%)	44 (19.2)	45 (22.0)	.558	0.068	41 (20.0)	45 (22.0)	.716	0.048
Comorbidities (%)								
Chronic liver disease	22 (9.6)	18 (8.8)	.896	0.029	22 (10.7)	18 (8.8)	.618	0.066
Chronic renal disease	4 (1.7)	3 (1.5)	1.000	0.023	4 (2.0)	3 (1.5)	1.000	0.038
Diabetes mellitus	22 (9.6)	19 (9.3)	.945	0.023	18 (8.8)	19 (9.3)	1.000	0.017
Sources of infection (%)								
Primary BSI	127 (55.5)	111 (54.1)	.859	0.026	117 (57.1)	111 (54.1)	.619	0.059
Pulmonary source of infection	29 (12.7)	31 (15.1)	.548	0.071	28 (13.7)	31 (15.1)	.778	0.042
Perianal infection	29 (12.7)	27 (13.2)	.989	0.015	26 (12.7)	27 (13.2)	1.000	0.015
Oral mucositis	33 (14.4)	22 (10.7)	.315	0.111	24 (11.7)	22 (10.7)	.876	0.031
Urinary tract	3 (1.3)	1 (0.5)	.695	0.08	3 (1.5)	1 (0.5)	.615	0.099
Skin and soft tissues	4 (1.7)	2 (1.0)	.783	0.06	4 (2.0)	2 (1.0)	.681	0.081
Abdominal	9 (3.9)	12 (5.9)	.479	0.08	9 (4.4)	12 (5.9)	.654	0.066
CRBSI	6 (2.6)	7 (3.4)	.839	0.04	5 (2.4)	7 (3.4)	.770	0.058
Infection source control (%)	220 (96.1)	197 (96.1)	1.000	0.001	196 (95.6)	197 (96.1)	1.000	0.024
Complications (%)								
Shock	6 (2.6)	9 (4.4)	.456	0.096	6 (2.9)	9 (4.4)	.599	0.078
Pneumonia	67 (29.3)	68 (33.2)	.438	0.085	63 (30.7)	68 (33.2)	.672	0.052
Oral mucositis	44 (21.5)	52 (22.7)	.845	0.030	43 (21.0)	44 (21.5)	1.000	0.012
Perianal mucositis	29 (12.7)	36 (17.6)	.196	0.137	29 (14.1)	36 (17.6)	.417	0.094
Day 1 ANC 0–500 cells/mL	220 (96.1)	194 (94.6)	.629	0.068	196 (95.6)	194 (94.6)	.819	0.045
Day 1 ANC 0–100 cells/mL	122 (53.3)	111 (54.1)	.932	0.017	110 (53.7)	111 (54.1)	1.000	0.010
Duration of neutropenia	10.0 (6.0–14.0)	11.0(4.0–17.0)	.362	0.085	10.0 (5.0–14.0)	10.0 (3.0–17.0)	.625	<0.001
IET48h	23 (10.0)	17 (8.3)	.643	0.061	20 (9.8)	17 (8.3)	.730	0.051
Combination antibiotic therapy								
β-Lactam + AG	62 (27.1)	64 (31.2)	.399	0.090	59 (28.8)	64 (31.2)	.666	0.053
CZA + AZT/AG	13 (5.7)	14 (6.8)	.766	0.040	13 (6.3)	14 (6.8)	1.000	0.020
MDR-PA	23 (10.0)	26 (12.7)	.474	0.083	23 (11.2)	26 (12.7)	.761	0.045
CRPA	38 (16.6)	46 (22.4)	.156	0.148	37 (18.0)	46 (22.4)	.325	0.109

Abbreviations: AG, Aminoglycoside; ALL, acute lymphoblastic leukemia; allo-HSCT, allogeneic hematologic stem-cell transplantation; AML, acute myeloid leukemia; ANC, absolute neutrophil counts; AZT, Aztreonam, β-lactams, beta-lactam antibiotics; BSI, bloodstream infection; CRPA, carbapenems-resistant *Pseudomonas aeruginosa*; CZA, Ceftazidime-Avibactam; Day 1 ANC 0–500 cells/mL, day at the onset of BSI; IET48h, inadequate empirical therapy within 48 h of the onset of PA BSI; IQR, interquartile range; MDR-PA, multidrug resistant *Pseudomonas aeruginosa*; Others, containing myelodysplastic syndrome (MDS) and lymphoma; PA, *Pseudomonas aeruginosa*; SMD, standardized mean difference.

In the weighted cohort, the univariate and multivariate analysis indicated that short course antibiotic therapy had similar outcomes to the prolonged course (Tables 2 and 3). The recurrent PA infection at any site or mortality within 30 days of completing therapy occurred in 8 (3.9%) patients in the short-course group and in 10 (4.9%) in the prolonged-course group (*P* = .979). The recurrent infection within 90 days occurred in 20 (9.8%) patients in the short-course group and in 13 (6.3%) patients in the prolonged-course group (*P* = .139), and the recurrent fever within 7 days occurred in 17 (8.3%) patients in the short-course group and in 15 (7.4%) in the prolonged-course group (*P* = .957). Multivariate analysis indicated that, excluding the duration of antibiotic treatment, MDR-PA, perianal mucositis, relapsed/refractory hematological malignancies and neutropenia non-recovery were the key determinants of recurrence and mortality within 30 days (Supplementary Table 4) (*P* < .05 for all).

**Table 2.** Univariate Analysis on the Clinical Outcomes of the Weighted Cohort

	Mortality or Recurrent Infection Within 30 D			Fever Relapse Within 7 D			Recurrent Infection Within 90 D		
Characteristic	No	Yes	*P* Value	No	Yes	*P* Value	No	Yes	*P* Value
Age, median (IQR), y	41.0 (26.0–51.5)	37.0 (27.0–52.0)	.809	42.0 (27.0–52.0)	32.0 (19.5–52.5)	.188	40.0 (27.0–52.0)	41.0 (26.0–51.0)	.897
Female (%)	179 (45.7)	8 (44.4)	.919	171 (45.4)	16 (48.5)	.729	170 (45.1)	17 (51.5)	.478
Disease (%)									
ALL	107 (27.3)	2 (11.1)	.129	104 (27.6)	5 (15.2)	.121	106 (28.1)	3 (9.1)	.**018**
AML	215 (54.8)	13 (72.2)	.147	208 (55.2)	20 (60.6)	.547	201 (53.3)	27 (81.8)	.**002**
Others	70 (17.9)	3 (16.7)	.897	65 (17.2)	8 (25.0)	.386	70 (18.6)	3 (9.1)	.260
Tumor stage (%)									
Induction	114 (29.1)	1 (5.6)	.030	103 (27.3)	12 (36.4)	.268	113 (30.0)	2 (6.1)	.**003**
Consolidation	198 (50.5)	6 (33.3)	.154	195 (51.7)	9 (27.3)	.**007**	185 (49.1)	19 (57.6)	.349
Relapse/refractory disease	80 (20.4)	11 (61.1)	**<**.**001**	80 (21.2)	11 (34.4)	.132	79 (21.0)	12 (36.4)	.068
Chemotherapy (%)	335 (85.5)	15 (83.3)	.803	323 (85.4)	27 (84.4)	1.000	320 (84.9)	30 (90.9)	.495
Allo-HSCT, past 100 d (%)	55 (14.0)	4 (22.2)	.333	54 (14.3)	5 (15.6)	1.000	54 (14.3)	5 (15.2)	1.000
Immunosuppressive therapy (%)	78 (19.9)	8 (44.4)	.**012**	76 (20.1)	10 (31.2)	.207	77 (20.4)	9 (27.3)	.482
Comorbidities (%)									
Chronic liver disease	39 (9.9)	1 (5.6)	.539	35 (9.3)	5 (15.2)	.276	39 (10.3)	1 (3.0)	.174
Chronic renal disease	23 (5.9)	1 (5.6)	.956	21 (5.6)	3 (9.4)	.623	22 (5.8)	2 (6.1)	1.000
Diabetes mellitus	34 (8.7)	3 (16.7)	.247	35 (9.3)	2 (6.1)	.535	34 (9.0)	3 (9.1)	.989
Sources of infection (%)									
Primary BSI	218 (55.6)	10 (55.6)	.996	210 (55.6)	18 (56.2)	1.000	211 (56.0)	17 (51.5)	.756
Pulmonary source of infection	57 (14.5)	2 (11.1)	.685	57 (15.1)	2 (6.2)	.270	55 (14.6)	4 (12.1)	.898
Perianal infection	50 (12.8)	3 (16.7)	.629	45 (11.9)	8 (25.0)	.065	46 (12.2)	7 (21.2)	.227
Complications (%)									
Shock	13 (3.3)	2 (11.1)	.085	14 (3.7)	1 (3.1)	1.000	13 (3.4)	2 (6.1)	.777
Pneumonia	122 (31.1)	9 (50.0)	.093	117 (31.0)	14 (43.8)	.196	114 (30.2)	17 (51.5)	.**020**
Perianal mucositis	59 (15.1)	6 (33.3)	.**038**	53 (14.0)	12 (37.5)	.**001**	56 (14.9)	9 (27.3)	.104
Day 1 ANC 0–100 cells/mL	208 (53.1)	13 (72.2)	.111	202 (53.6)	19 (57.6)	.659	202 (53.3)	19 (61.3)	.391
Duration of neutropenia, median (IQR)	10.0 (4.0–15.0)	10.5 (6.0–22.5)	.391	10.0 (4.0–15.0)	10.0 (5.0–20.0)	.531	10.0 (4.0–15.0)	11.0 (8.0–20.0)	.095
IET48h	34 (8.7)	3 (16.7)	.247	33 (8.7)	4 (12.5)	.694	31 (8.2)	6 (18.2)	.110
ANC 0–500 cells/mL at the day of discontinuation of antibiotics	39 (9.9)	8 (44.4)	**<**.**001**	36 (9.5)	11 (34.4)	**<**.**001**	38 (10.1)	9 (27.3)	.**007**
Monotherapy	209 (53.3)	6 (33.3)	.156	199 (52.6)	16 (50.0)	.918	200 (53.1)	15 (45.5)	.512
MDR-PA	42 (10.7)	7 (38.9)	**<**.**001**	41 (10.8)	8 (25.0)	.**037**	39 (10.3)	10 (30.3)	.**002**
CRPA	76 (19.4)	7 (38.9)	.**044**	74 (19.6)	9 (28.1)	.354	73 (19.4)	10 (30.3)	.203
Short course antibiotic therapy	197 (50.3)	8 (44.4)	.630	190 (50.3)	15 (46.9)	.854	186 (49.3)	19 (57.6)	.468

Abbreviations: ALL, acute lymphoblastic leukemia; allo-HSCT, allogeneic hematologic stem-cell transplantation; AML, acute myeloid leukemia; ANC, absolute neutrophil counts; BSI, bloodstream infection; CRPA, carbapenems-resistant *Pseudomonas aeruginosa*; CZA, Ceftazidime-Avibactam; Day 1 ANC 0–500 cells/mL, day at the onset of BSI; IET48h, inadequate empirical therapy within 48 h of the onset of PA BSI; IQR, interquartile range; MDR-PA, multidrug resistant *Pseudomonas aeruginosa*; Others, containing myelodysplastic syndrome (MDS) and lymphoma; PA, *Pseudomonas aeruginosa*; SMD, standardized mean difference. Values in bold means *P* < .05.

**Table 3.** Multivariate Analysis on the Clinical Outcomes in the Weighted Cohort

Characteristic	Mortality or Recurrent Infection Within 30 D		Fever Relapse Within 7 D		Recurrent Infection Within 90 D	
	aOR (95% CI)	*P*	aOR (95% CI)	*P*	aOR (95% CI)	*P*
Short course treatment	…	.979	…	.957	…	.139

Abbreviations: aOR, adjusted odds ratio; CI, confidence interval.

On average, patients who received short-course therapy spent 3.3 fewer days in the hospital compared to patients who received longer courses (3.31 days, 95% CI: 3.48 days, 13.74 days, *P* < .001). The median hospital days were 32.01 days in the short-course group and 40.62 days in the prolonged-course group.

## DISCUSSION

This study, by including patients with hematological malignancies and balancing confounding factors through IPTW, yielded results indicating that there was no difference in fever relapse within 7 days, death or recurrent infection within 30 days, and recurrent infection within 90 days of completing antibiotic therapy regardless of whether patients were treated with a short course (median, 8 days) or prolonged course (median, 15 days) of antibiotics. Furthermore, patients treated with shorter courses were discharged from the hospital approximately 3.3 days sooner than those who received prolonged course of treatment.

Although current guidelines recommend antibiotic treatment for PA BSI lasting more than 14 days [1], some studies in immunocompetent populations have already indicated that short-course therapy is non-inferior to prolonged treatment in terms of clinical and microbiological cure rates. Siddharth Swamy et al [15] compared the effectiveness of short-course and prolonged-course treatments in a retrospective analysis of 178 patients with gram-negative BSI and found that short-course therapy for gram-negative bacteremia yielded comparable rates of clinical response (78.6% vs 80.6%, *P* = .202) and microbiological cure (83.3% vs 91.7%, *P* = .690). Rodrigues et al [16] indicated that short-term therapy was not associated with 30-day mortality(hazard ratio [HR] = 1.01, 95% CI .47–2.20, *P* = .98), Bae et al [17] included 290 uncomplicated PA BSI and found that no significant difference in the risk of recurrence or 30 day mortality between the prolonged-course and short-course groups (HR = 0.68, 95% CI = .34–1.36, *P* = .28) and the recurrence of PA infection within 180 days (HR = 0.57, 95% CI = .29–1.10, *P* = .09). However, several retrospective studies involving immunocompromised populations are as follows. A study by Metais et al [18] included 71 AML patients with 104 BSI episodes, of which 48 (46%) received short-course treatment. Only 8 (7.6%) BSI episodes relapsed within 30 days after discontinuation of antibiotics, with 5 of them having received short-course treatment. No significant association was found between the risk of relapse and short-course antibiotic treatment (*P* = .37). This study has limitations, including a small sample size, a mix of microbial infections comprising 42%, with gram-negative infections accounting for only 15%. In a study by Fabre et al [19], they included 249 patients with PA BSI for matched analysis. The authors claimed that 65% of the patients were immunocompromised, but only 23% had recent chemotherapy and 8% had allo-HSCT within a year, which does not reflect the reality in hematology wards. The sample size was also small, especially with only 72 cases in the short-course group, making it unclear if this treatment was safe for immunocompromised patients. Our study addresses this gap by including multicenter onco-hematology patients. To enhance the validity and generalizability of our research, we applied stringent inclusion and exclusion criteria. We categorized the antibiotic duration into short course (7–11 days) and prolonged course (12–21 days) and excluded patients who received antibiotics outside this range to eliminate the confounding effect of inadequate or excessive antibiotic therapy. We also excluded patients who had death or other events before completing the antibiotic course to avoid the immortal time bias. Furthermore, we excluded patients who developed septic shock early in the course of infection, as they may have different antibiotic requirements and outcomes than other patients. These criteria minimized potential biases and confounders and approximated our study to the real-world scenario.

Our cohort study included patients who regularly received treatment and follow-up at our center and had follow-up information on mortality and recurrence for 30 and 90 days after antibiotic therapy, which facilitating the accurate assessment of the short-term and long-term effects of antibiotic duration on infection outcomes. This made our study more comparable to the clinical trials and the results more reliable. Furthermore, the variables used in the weighted analysis were known major prognostic factors and can be acquired at the onset of BSI, which closely resembled the standard of randomization in RCTs. Our study population had a relatively low prevalence of comorbidities due to our populations being predominantly young, akin to the features of BSI in hematological patients [20]. While regarding the sources of BSI, we observed that over 50% of cases were primary BSI, indicating the absence of signs of infection at other sites. Among patients in whom the source could be determined, pulmonary and the damaged mucosal including oral and perianal were the main sites, whereas the CRBSI and urinary tract were quite rare in our study. These findings deviated from previous studies reporting that gram-negative BSIs primarily originated from venous catheters and urinary sources [19]. Those studies often involved intensive care unit (ICU) populations with a higher prevalence of invasive devices such as urinary catheters and central lines. Although the CRBSI rate was relatively low, which might be related to the use of a fixed catheterization room, and the standardized daily care. However, it was also related to the fact that some patients did not achieve bilateral double suction, thus failing to meet the diagnostic criteria for CRBSI. This is also an area that needs to be improved in infection control in the future. In total, our study revealed the main characteristics of PA BSI in patients with hematologic malignancies, which were more consistent with actual infection patterns compared to previously published researches. Although a very small number of patients did not completely recover from neutropenia when they stopped antibiotics, they had an upward trend in the ANC and were evenly distributed in both short-course and long-course groups. The neutropenia non-recovery populations had worse clinical outcomes, which warrant physicians to take measures other than prolonged the antibiotic duration.

Based on our real-world and retrospective study, short course antibiotic therapy is effective for PA BSI in part of hematologic malignancy patients and may have some potential benefits, such as reducing the exposure to antibiotics, lowering the cost of treatment and shortening hospital stays. The primary factors influencing recurrence and mortality were identified as MDR bacterial infections, perianal or pulmonary infections, and persistent or recurrent hematological malignancies. These findings highlight the importance of clinical attention to these factors in order to improve patient outcomes.

Our study also provides robust clinical evidence to support future RCTs. Onco-hematological patients are often excluded from RCTs [21], limiting the availability of compelling evidence. By utilizing a large-scale cohort matched analysis, our study demonstrates the comparable safety of short-course treatment, thus serving as a crucial foundation for the implementation of RCTs in clinical practice. Notably, onco-hematological patients at a lower risk of poor outcomes, including those showing rapid clinical improvement after antibiotic initiation, and those with milder levels of immunosuppression, should be given priority in RCTs enrollment. Clinical guidelines should also provide specific recommendations regarding antibiotic duration for immunocompromised patients, taking into account the potential for shorter treatment durations.

Given the retrospective nature of our study, certain limitations should be acknowledged. First, we screened a subset of patients into the final study through inclusion and exclusion criteria. Although our intention was to better evaluate the indicators and avoid immortal time bias, this also made our study results not generalizable to all hematological malignancy patients. Second, there was randomness in the timing of repeating blood cultures after initiating targeted antibiotic therapy by the physicians in our study, which is a problem faced by many studies [22], as it is impossible to achieve fixed-time re-examination assembled in clinical trials. This may lead to bias in decision making of antibiotic duration. Further RCTs are necessary to validate the reproducibility of our findings.

In conclusion, we found that short course of antibiotic therapy was not inferior to the prolonged course of therapy in part of onco-hematological patients with PA BSI in our study, with the potential added benefit of earlier hospital discharge if treated with a shorter course.

## Supplementary Data

Supplementary materials are available at *Clinical Infectious Diseases* online. Consisting of data provided by the authors to benefit the reader, the posted materials are not copyedited and are the sole responsibility of the authors, so questions or comments should be addressed to the corresponding author.

## Supplementary Material

ciad605_Supplementary_Data

## Notes

***Author Contributions.*** X. F. was responsible for the data curation methodology and writing the original draft. C. Q., J. W., J. L., and Q. L. contributed to the data collection. Y. F. contributed to the data collection and writing and editing. E. J., Y. M., L. Q., Z. X., and J. W. finished the formal analysis and supervision. S. F. and M. H. supplied the conceptualization, funding acquisition, resources, supervision, and writing—review and editing. All authors reviewed the manuscript.

***Acknowledgments***. The authors would like to thank all the reviewers who participated in the review, as well as for providing English editing services during the preparation of this manuscript.

***Ethics approval and consent to participate.*** This study was approved by the Institutional Review Board and Ethics Committee of the Institute of Hematology and Blood Diseases Hospital. The ethics committee approved the waiver of informed consent owing to the retrospective nature of the review. All human data of patients’ records were confirmed for collection in accordance with the relevant guidelines and regulations.

***Financial support*.** This work was supported by the Chinese Academy of Medical Sciences Innovation Fund for Medical Sciences (grant numbers 2021-I2M-1-017; 2021-I2M-C&T-B-080), Tianjin Municipal Science and Technology Commission Grant (grant number 21JCZDJC01170) and Haihe Laboratory of Cell Ecosystem Innovation Fund (grant number 22HHXBSS00036).
