# Supplementary material for: Is Short-Course Antibiotic Therapy Suitable for Pseudomonas aeruginosa Bloodstream Infections in Onco-hematology Patients With Febrile Neutropenia? Results of a Multi-institutional Analysis
Source: Clin Infect Dis. 2023 Oct 5;78(3):518–25. doi: 10.1093/cid/ciad605 (PMC10954337; doi:10.1093/cid/ciad605)
Supplement: ciad605_Supplementary_Data [file ciad605_supplementary_data.docx]

**Supplemental material**

**Methods**

**Study design**

Our practice for repeating blood cultures during antibiotic treatment involves specific situations^1-3^:

- When patients continue to exhibit fever, or signs of infection 72 hours after initiating definite antibiotic therapy, repeat blood culture to rule out relapse or persistent infection.

- When patients have normal body temperature for more than 72 hours, resolution of infection-related symptoms, and normalization of inflammatory markers after antibiotic treatment, and the clinician intends to discontinue treatment, repeat blood culture to confirm pathogen clearance.

Most of patients experienced at least one-time repeating blood culture during the antibiotic treatment.

While recognizing the variability in timing of follow-up blood cultures across different physicians due to the absence of clear evidence supporting a fixed course of antibiotic treatment for bloodstream infections in hematological patients, we acknowledge that individual medical experience could influence the time to repeat the blood culture.

**Table S1 Definitions of the source of infection**

| **Source of infection** | **Definitions** |
| --- | --- |
| **Primary BSI** | A Laboratory Confirmed Bloodstream Infection (LCBI) that is not secondary to an infection at another body site  (In this study, mainly are mucosal barrier injury laboratory-confirmed bloodstream Infection due to the chemotherapy or HSCT.) |
| **Secondary BSI** |  |
| Pulmonary source of infection | - Symptoms of lower respiratory tract infection; - pulmonary infiltrates on CT scans; - the isolation of PA in a qualified sputum specimen or bronchoalveolar lavage fluid, but the latter is not mandatory; - without any other potential source of bacteremia; |
| Perianal infection | - Symptoms of perianal erythema, swelling, tenderness, and itching. - Swab screening of the perianal area confirms the presence of PA |
| oral mucositis | - oral mucosal redness, ulceration, rupture, and gingival pain; - A swab screening of the oral-pharyngeal area also reveals the presence of PA |
| Intra-abdominal | - Digestive symptoms (diarrhea, intense abdominal pain, severe mucositis) - Radiological signs of GI inflammation or infection (colitis, abscess) |
| Urinary tract | - The symptoms of urinary tract infection include frequency, urgency, dysuria, discomfort during urination, lower abdominal pain, and in some cases, a rapid onset of difficulty in urination. - Pus in urine or isolation of PA from urine culture. - Control measures include implementing antimicrobial measures or removing the catheter. |
| Skin and soft  tissues | Skin abscesses, furuncles, carbuncles and inflamed epidermoid cysts, erysipelas, cellulitis, necrotizing fasciitis, surgical site infections |
| CRBSI | presence of symptoms consistent with BSI related to the catheter, along with the detection of catheter-related pathogens in blood cultures. The cessation of clinical symptoms after the removal of the catheter was determined as the control of infection. |

**Table S2 Clinical characteristics of the cohort before exclusion (n=694)**

|  | survivors  (n=615, 88.6%) | Non-survivors  (n=79, 11.4%) | p |
| --- | --- | --- | --- |
| **Age, median (IQR), y** | 40.0 (27.0-51.0) | 46.0 (33.0-55.0) | **0.011** |
| **Female (%)** | 279 (45.4) | 23 (29.1) | **0.009** |
| **Disease (%)** |  |  | 0.449 |
| ALL | 145 (23.6) | 22 (27.8) |  |
| AML | 365 (59.3) | 41 (51.9) |  |
| Others | 105 (17.1) | 16 (20.3) |  |
| **Tumor stage (%)** |  |  | **<0.001** |
| Induction | 154 (25.0) | 27 (34.2) |  |
| Consolidation | 336 (54.6) | 20 (25.3) |  |
| salvage | 125 (20.3) | 32 (40.5) |  |
| **Chemotherapy (%)** | 530 (86.2) | 67 (84.8) | 0.875 |
| **Allo-HSCT, past 100 days (%)** | 76 (12.4) | 6 (7.6) | 0.294 |
| **Immunosuppressive therapy (%)** | 136 (22.1) | 12 (15.2) | 0.205 |
| **Comorbidities (%)** |  |  | 0.564 |
| Chronic liver disease | 53 (8.6) | 5 (6.3) |  |
| Chronic renal disease | 9 (1.5) | 4 (5.0) |  |
| Diabetes mellitus | 50 (8.1) | 8 (10.1) |  |
| **Sources of infection (%)** |  |  | 0.179 |
| Primary BSI | 348 (56.6) | 35 (44.3) |  |
| Pulmonary source of infection | 87 (14.1) | 20 (25.3) |  |
| Perianal infection | 67 (10.9) | 16 (20.3) |  |
| Oral mucositis | 88 (14.3) | 8 (10.1) |  |
| Urine tract | 5 (0.8) | 1 (1.3) |  |
| Skin and soft  tissues | 6 (1.0) | 1 (1.3) |  |
| Abdominal | 30 (4.9) | 5 (6.3) |  |
| CRBSI | 13 (2.1) | 0 |  |
| **Infection control (%)** | 592 (96.3) | 66 (83.5) | **<0.001** |
| **Complications (%)** |  |  | **<0.001** |
| Shock | 20 (3.3) | 29 (36.7) |  |
| Pneumonia | 185 (30.1) | 61 (77.2) |  |
| oral mucositis | 140 (22.8) | 19 (24.1) |  |
| Perianal mucositis | 86 (14.0) | 18 (22.8) |  |
| **Day 1 absolute neutrophil count 0–100 cells/mL (%)** | 318 (51.7) | 47 (59.5) | 0.236 |
| **Duration of neutropenia (median [IQR])** | 9.0 (4.0-15.0) | 8.0 (3.0-20.0) | 0.910 |
| **Duration of antibiotic treatment (median [IQR])** | 10.0 (7.0-15.0) | 10.0 (4.0-18.0) | 0.174 |
| **IET48h (%)** | 48 (7.8) | 37 (46.8) | **<0.001** |
| **Combination antibiotic therapy (%)** |  |  | **<0.001** |
| β-Lactam+AG | 160 (26.0) | 26 (32.9) |  |
| CZA+AZT/AG | 37 (6.0) | 12 (15.2) |  |
| **MDR-PA (%)** | 63 (10.2) | 27 (34.2) | **<0.001** |
| **CRPA (%)** | 103 (16.7) | 34 (43.0) | **<0.001** |

**Table S3 The distribution of the populations who didn’t have neutropenia recovery at the end of antibiotics treatment**

| **Characteristic** | **Full cohort** | | | | **Weighted cohort** | | | |
| --- | --- | --- | --- | --- | --- | --- | --- | --- |
|  | **Short course** | **Prolonged course** | **P value** | **SMD** | **Short course** | **Prolonged course** | **P value** | **SMD** |
| **ANC 0–500 cells/mL at the day of discontinuation of antibiotics (%)** | 24 (10.5) | 24 (11.7) | 0.950 | 0.180 | 24 (11.7) | 23 (11.2) | **0.980** | **0.090** |
| **Clinical outcomes of the neutropenia non-recovery** | | | | | | | | |
| **Fever relapse within 7 days (%)** | 8 (33.3) | 5(20.8) | 0.390 | 0.540 | 7 (29.2) | 4 (17.4) | 0.430 | 0.490 |
| **Mortality or recurrent infection within 30 days (%)** | 4 (16.7) | 4 (16.7) | 1.000 | 0.000 | 4 (16.7) | 4 (17.4) | 1.000 | 0.030 |
| **Recurrent infection within 90 days (%)** | 5 (20.8) | 4 (16.7) | 0.780 | 0.170 | 5 (20.8) | 4 (17.4) | 0.840 | 0.140 |

**Table S4 Multivariate analysis of the weighted cohort**

| **Characteristic** | **Mortality or recurrent infection within 30 days** | | **Fever relapse within 7 days** | | **Recurrent infection within 90 days** | |
| --- | --- | --- | --- | --- | --- | --- |
|  | **aOR (95%CI)** | ***p*** | **aOR (95%CI)** | ***p*** | **aOR (95%CI)** | ***p*** |
| **Short course treatment** | - | 0.979 | - | 0.957 | - | 0.139 |
| **MDR-PA** | 10.825 (3.106-37.733) | <0.001 | 3.990 (1.521-10.468) | 0.005 | 5.011 (2.073-12.113) | <0.001 |
| **Relapse/refractory disease** | 8.756 (2.802-27.366) | <0.001 | - | 0.071 | 2.382 (1.067-5.321) | 0.034 |
| **shock** | - | 0.066 | - | 0.912 | - | 0.456 |
| **Pneumonia** | - | 0.140 | - | 0.402 | 2.566 (1.177-5.595) | 0.018 |
| **Perianal mucositis** | 7.875 (2.146-28.891) | 0.002 | 5.652 (2.370-13.475) | <0.001 | - | 0.051 |
| **ANC 0–500 cells/mL at the day of discontinuation of antibiotics** | 13.983 (4.022-48.617) | <0.001 | 7.562 (3.060-18.685) | <0.001 | 4.056 (1.624-10.128) | 0.003 |

1. Chela HK, Vasudevan A, Rojas-Moreno C, Naqvi SH. Approach to Positive Blood Cultures in the Hospitalized Patient: A Rev iew. *Missouri medicine*;116(4):313-317.

2. Canzoneri CN, Akhavan BJ, Tosur Z, Andrade PEA, Aisenberg GM. Follow-up Blood Cultures in Gram-Negative Bacteremia: Are They Needed? *Clinical infectious diseases : an official publication of the Infectio us Diseases Society of America*;65(11):1776-1779.

3. Mushtaq A, Bredell BX, Soubani AO. Repeating blood cultures after initial bacteremia: When and how often? *Cleveland Clinic journal of medicine*;86(2):89-92.
